# Outcomes in patients with chronic heart failure undergoing non‐cardiac surgery: a secondary analysis of the METREPAIR international cohort study*

**DOI:** 10.1111/anae.16607

**Published:** 2025-04-15

**Authors:** Anna Kirkopoulos, René M'Pembele, Sebastian Roth, Alexandra Stroda, Jan Larmann, Hans‐Joerg Gillmann, Katarzyna Kotfis, Michael T. Ganter, Daniel Bolliger, Miodrag Filipovic, Luca Guzzetti, Eckhard Mauermann, Daniela Ionescu, Savino Spadaro, Wojciech Szczeklik, Stefan De Hert, Beatrice Beck‐Schimmer, Simon J. Howell, Giovanna A. Lurati Buse, METREPAIR Investigators, Purificación Matute, Sanem Cakar Turhan, Judith van Waes, Filipa Lagarto, Kassiani Theodoraki, Anil Gupta, Alexey Ovezov, Biljana Kuzmanovska, Stjepan Barisin, Peter Poredos, Daniela Arabadzhieva, Dragana Unic‐Stojanovic, Edith Fleischmann, Claude Meistelman, Donal J. Buggy, Paul Calleja, Antigona Hasani Sebastian Roth, René M'Pembele, Johannes Nienhaus, Alexandra Stroda, Theresa Tenge, Anna Kirkopoulos, Ragnar Huhn, Detlef Kindgen‐Milles, Cornelia Schultze, Nele Verbarg, Christian Gehrke, Anna Katharina Klemann, Friederike Hagebölling, Hans‐Jörg Gillmann, Svenja Albrecht, Jakob Stroeder, Ann‐Kristin Schubert, Hinnerk Wulf, Florian Espeter, Benedikt Russe, Jan Larmann, Markus A. Weigand, Lars Bergmann, Matthias Unterberg, Petra Bischoff, Raphael Pirzer, Patric Rene Rach, Klaus Ott, Alexander Zarbock, Ana Kowark, Claudia Neumann, Bahareh Marchand, Christoph Sponholz, Henrik Rueffert, Mira Kramer, Simone Lindau, Patrick Meybohm, Kai Zacharowski, Götz Schmidt, Christian Koch, Melissa Carollo, Cecilia Novazzi, Fiorenza Toso, Alessandro Bacuzzi, Luca Guzzetti, Riccardo Ragazzi, Carlo Alberto Volta, Francesco De Giorgi, Barbara Bacer, Antonio Federico, Davide Chiumello, Luigi Vetrugno, Alberto Castella, Simonetta Tesoro, Antonella Cotoia, Elena Bignami, Agrippino Bellissima, Andrea Cortegiani, Marco Crisman, Arturo Toninelli, Ornella Piazza, Lucia Mirabella, Matteo Bossolasco, Francesco Bona, Juan Manuel Perdomo, Miquel Coca‐Martinez, Albert Carramiñana, Marta Giné Servén, Astrid Batalla González, José Maria Gil Sánchez, Ángel Becerra‐Bolaños, Aurelio Rodríguez‐Pérez, Anna Artigas Soler, Morena Basso, Anna Peig Font, Diana Vernetta, Julia Hernando Santos, Enrique Alday Muñoz, Mercedes Cabellos Olivares, Gregorio Marco, Maria Bermudez Lopez, Javier Barrio, María Isabel Forés, Estefanía Boix, Mercedes Ayuso, Bogdan Sorel Petre, Ioana Sorina Oprea, Mihai Dan Latiș, Dan Corneci, Simona Margarit, Horatiu Vasian, Dana Tomescu, Iulia Cîndea, Dan Sebastian Dirzu, Sanda‐Maria Copotoiu, Alida Moise, Serban Bubenek‐Turconi, Liana Valeanu, Patrick Mark Wanner, Mirjana Djurdjevic, Sandra Nuth, Miodrag Filipovic, Esther Seeberger, Nicolai Goettel, Firmin Kamber, Eckhard Mauermann, Frederique Chammartin, Michael Thomas Ganter, Thomas Jan Gerber, Daniela Schneebeli, Andreas Pregernig, Beatrice Beck‐Schimmer, Sina Grape, Simon Tomala, Bernardo Bollen Pinto, Maciej Żukowski, Małgorzata Zegan‐Barańska, Igor Karolak, Katarzyna Kotfis, Lukasz Krzych, Szymon Czajka, Dorota Studzińska, Anna Kluzik, Tomasz Koszel, Izabela Pabjańczyk, Anna Gajdosz, Suheyla Karadag Erkoc, Basak Ceyda Meco, Ahmet Kemalettin Koltka, Muserref Beril Dincer, Perihan Ekmekçi, Kemal Tolga Saracoglu, Filiz Alkaya Solmaz, Menekse Ozcelik, Oguzhan Arun, Ozlem Korkmaz Dilmen, Benedikt Preckel, Markus W. Hollmann, Yannick Hazen, Hans Donald de Boer, Anne Epema, Seppe Koopman, Felix Van Lier, Rita Pinto, André Carrão, Daniel Ribeiro, Joana Mourão, Miguel Coelho, Nuno Losa, Nuno Santos, Luis Cabral, Diana Afonso, Sérgio Zenha, Cristina Ramos, Carla Hipólito, Maria Vasilaki, Antonia Andreeva, Donika Zaimi, Athanasios Chalkias, Maria Spyraki, Martina Rekatsina, Georgia Tsaousi, Anthony Short, Sonja Meier, Thumuluru Kavitha Madhuri, Scott Latham, James Knock, Andrew Drummond, Fiona Ramsden, Kubisz‐Pudelko, James Walker, Iain Moppett, Louise White, Simon J. Howell, Matthew Jackson, Henrik Reschreiter, Richard Innes, Michelle Chew, Sigridur Kalman, Jakob Wallden, Anna Schening, Lina Jonikaite, Anna Enlund, Luc De Baerdemaeker, Stuart Morrison, Steffen Rex, Alexandros Alexis, Viktoria E. Khoronenko, Vladislav Belskii, Kseniya Kaznacheeva, Alexey Gritsan, Liljana Malinovska‐Nikolovska, Mladjan Golubović, Oskar Kotzinger, Marc Danguy Des Deserts, Nicolas Ducrocq, Jean François Bonnet, Barbara Cusack, Antigona Hasani, Rajmonda Nallbani, Sylvia Daamen, Benoit Plichon, Pierre Harlet, Slama Farsi, Saman Homayun Sepehr, David Espinosa

**Affiliations:** ^1^ Anesthesiology Department University Hospital Düsseldorf, Heinrich Heine University Düsseldorf Germany; ^2^ Department of Anesthesiology Heidelberg University Hospital Heidelberg Germany; ^3^ Department of Anaesthesiology and Intensive Care Medicine Hannover Medical School Hannover Germany; ^4^ Department of Anesthesiology, Intensive Care and Pain Management Pomeranian Medical University Szczecin Poland; ^5^ Department of Anesthesiology Kantonsspital Winterthur Winterthur Switzerland; ^6^ Clinic for Anaesthesia, Intermediate Care, Prehospital Emergency Medicine and Pain Therapy University Hospital Basel Basel Switzerland; ^7^ Division of Perioperative Intensive Care Medicine Kantonsspital St Gallen St Gallen Switzerland; ^8^ Anesthesia and Intensive Care Department University Hospital Varese Italy; ^9^ Department of Anesthesiology Zurich City Hospital Zurich Switzerland; ^10^ Department of Anaesthesia and Intensive Care I Iuliu Hatieganu University of Medicine and Pharmacy Cluj‐Napoca Romania; ^11^ Department of Translational Medicine University of Ferrara Ferrara Italy; ^12^ Center for Intensive Care and Perioperative Medicine, Jagiellonian University Medical College Kraków Poland; ^13^ Department of Anaesthesiology and Peri‐operative Medicine Ghent University Hospital, Ghent University Ghent Belgium; ^14^ Institute of Anaesthesiology University Hospital Zurich, University of Zurich Zurich Switzerland; ^15^ Leeds Institute of Medical Research at St James's, University of Leeds Leeds UK

**Keywords:** cardiovascular risk, guideline adherence, mortality, postoperative complications

## Abstract

**Introduction:**

Heart failure is a frequent comorbidity in patients undergoing non‐cardiac surgery and an acknowledged risk factor for postoperative mortality. The associations between stable chronic heart failure and postoperative outcomes have not been explored extensively. The aim of this study was to determine associations between stable chronic heart failure and its peri‐operative management and postoperative outcomes after major non‐cardiac surgery.

**Methods:**

This is a secondary analysis of MET‐REPAIR, an international prospective cohort study including patients undergoing non‐cardiac surgery aged ≥ 45 y with increased cardiovascular risk. Main exposures were stable chronic heart failure and availability of a pre‐operative transthoracic echocardiogram. The primary endpoint was the incidence of postoperative major adverse cardiovascular events at 30 days. Secondary endpoints included 30‐day mortality and severe in‐hospital complications. Multivariable logistic regression models were calculated.

**Results:**

Of 15,158 included patients, 3880 (25.6%) fulfilled the diagnostic criteria for stable chronic heart failure, of whom 1397 (36%) were female. Chronic heart failure was associated with increased risk of postoperative 30‐day major adverse cardiovascular events (OR 2.04, 95%CI 1.59–2.60), 30‐day mortality (OR 1.50, 95%CI 1.17–1.92) and in‐hospital complications (OR 1.47, 95%CI 1.30–1.66). Transthoracic echocardiography was performed in 1267 (32.7%) patients with heart failure; 146 (11.5%) patients with heart failure presented with a left ventricular ejection fraction < 40%. Reduced ejection fraction was associated with major adverse cardiovascular events (OR 2.0, 95%CI 1.01–3.81).

**Discussion:**

Stable chronic heart failure is independently associated with major adverse cardiovascular events, mortality and severe postoperative complications when measured 30 days after non‐cardiac surgery.

## Introduction

Heart failure is a relatively common condition that affects 17 of 1000 people in Europe and its prevalence is increasing [1]. Due to advances in heart failure treatment, patients survive longer and frequently develop non‐cardiac disease that requires surgical treatment. Such patients are at increased risk of postoperative complications [2, 3, 4]. Acutely symptomatic or worsening heart failure, as well as a previous history of congestive heart failure, are well‐established risk factors for postoperative mortality and morbidity after non‐cardiac surgery [5, 6, 7, 8]. While data from American administrative databases suggest that even patients with asymptomatic heart failure had poorer survival at 90 days compared with patients without heart failure after non‐cardiac surgery [9], the prognostic impact of stable heart failure in patients undergoing non‐cardiac surgery is less well established than it is for acute, worsening or symptomatic heart failure.

The 2022 European Society of Cardiology guidelines on cardiovascular assessment and management of patients undergoing non‐cardiac surgery [10] recommend pre‐operative transthoracic echocardiography (TTE) in patients with poor functional capacity, high N‐type pro brain natriuretic peptide (NT‐proBNP, ≥ 125 pg.ml^‐1^) or valvular disease before high‐risk non‐cardiac surgery (class 1 recommendation). It is not known to what extent this recommendation is implemented in clinical practice and how many patients with elevated cardiac risk receive a pre‐operative TTE before non‐cardiac surgery.

The aim of this secondary analysis was to quantify the associations between chronic stable heart failure and postoperative outcomes including major adverse cardiovascular events (MACE), mortality and in‐hospital complications in a large European sample of patients having non‐cardiac surgery. We also aimed to describe peri‐operative management in terms of pre‐operative echocardiography and the association between heart failure severity by left ventricular ejection fraction with MACE, mortality and in‐hospital complications at 30 days after surgery.

## Methods

This is a secondary analysis of the MET‐REPAIR study (NCT03016936), an international prospective cohort study that enrolled patients in 150 centres in 25 European countries between June 2017 and April 2020 [11]. Ethical approval was obtained in all participating centres before enrolment, and patients provided informed consent where it was required [11]. The parent study evaluated the association between self‐reported effort tolerance and postoperative outcomes as well as the predictive value of self‐reported functional capacity when added to clinical risk factors [11] (protocol available on the European Society of Anaesthesiology website [12]). Ethical approval for this secondary analysis was obtained at Heinrich Heine University Duesseldorf. This work is reported according to STROBE guidelines [13].

Patients undergoing elective in‐hospital non‐cardiac surgery were eligible if aged > 45 y with a Revised Cardiac Risk Index (RCRI) ≥ 2 [7]; or a National Surgical Quality Improvement Program Risk Calculator for Myocardial Infarction and Cardiac Arrest (NSQIP MICA) > 1% [14]; or if aged > 65 y and planned for moderate or high‐risk procedures [15]. Surgical interventions can be categorised by their estimated 30‐day cardiac event rates into low‐ (< 1%), moderate‐ (1–5%) or high‐risk (> 5%) groups [15]. Patients with acute coronary syndrome or uncontrolled congestive heart failure (defined as acutely decompensated heart failure with signs of congestion such as pulmonary oedema, acute respiratory failure, dyspnoea, hypotension or cardiogenic shock [16]) in the preceding 30 days or stroke in the 7 days before surgery were not studied.

The primary endpoint of this analysis was the incidence of MACE measured 30 days after surgery; MACE was defined as a composite of intra‐ or postoperative cardiovascular mortality; non‐fatal cardiac arrest; acute myocardial infarction (according to the third universal definition of myocardial infarction [17]); stroke or transient ischaemic attack; and/or worsening of congestive heart failure requiring transfer to a higher unit of care or prolonged stay in the intensive care unit or intermediate care (lasting ≥ 24 h). Secondary endpoints were 30‐day all‐cause mortality and in‐hospital severe postoperative complications defined by a Clavien‐Dindo classification ≥ 3 [18]. Local investigators adjudicated the events based on the criteria defined in the parent study [11].

The main explanatory variable was pre‐operative stable chronic heart failure as defined according to current guidelines [16] by either: previous episodes of congestive heart failure; left ventricular ejection fraction < 50%; or an elevated NT‐proBNP concentration > 125 pg/ml^‐1^ in the presence of heart failure symptoms (breathlessness, orthopnoea, paroxysmal nocturnal dyspnoea, ankle swelling, fatigue [16]). This definition is in keeping with current guidelines [16] and was employed to include as many patients with heart failure as possible. Using this definition, prevalence and characteristics in patients with heart failure were analysed and compared with patients without heart failure. Of note, the parent study excluded patients with uncontrolled heart failure in the last 30 days before non‐cardiac surgery [11].

For analysis of peri‐operative heart failure management, a subgroup with available left ventricular ejection fraction data was defined. Herein, heart failure was classified as heart failure with preserved (HFpEF; left ventricular ejection fraction ≥ 50%); mildly reduced (HFmrEF; left ventricular ejection fraction 40–49%) and reduced (HFrEF, left ventricular ejection fraction < 40%) ejection fraction [19].

Data for explanatory and covariables were extracted from medical records by trained personnel for the prospective parent study. Statistical analysis was performed using IBM SPSS© software version 29 (Armonk, NY, USA). Normality was tested with the Shapiro–Wilk test and Q–Q plots. For all statistical tests, p < 0.05 was considered significant.

To analyse the impact of heart failure and its peri‐operative management on postoperative MACE, mortality and severe in‐hospital complications, we conducted multivariable logistic regression analyses with forced entry of predefined variables. These were specifically: age; sex; ASA physical status (dichotomised at ≥ 3); estimated glomerular filtration rate (eGFR, categorised as < 30 ml.min^‐1^ or dialysis, 30–60 ml.min^‐1^ or ≥ 60 ml.min^‐1^); active cancer; type of surgery (low, moderate or high‐risk procedures) [16]; diabetes mellitus; hypertension; coronary artery disease; chronic obstructive pulmonary disease; peripheral vascular disease; and stroke. Covariates were defined and categorised a priori [7, 11, 14, 20] (online Supporting Information Table S1). All the included covariables for multivariable logistic regression analysis are established predictors for MACE after non‐cardiac surgery. We decided against employing RCRI or NSQIP MICA primarily as covariables in this analysis because the endpoint for which those scores were developed and validated differs from the study endpoint in this analysis [7, 14], and because of the limited discrimination of RCRI in previous studies [8, 21].

For the explanatory analysis of the subsample with TTE data, left ventricular ejection fraction was categorised as preserved (≥ 50%), mildly reduced (40–49%) or reduced (< 40%) according to ESC guidelines [19]. Due to the more limited number of events, for this analysis, RCRI [7] and age only were used for adjustment.

## Results

Of 15,158 patients, 15,767 patients (96.1%) had complete 30‐day follow‐up. From these, 3880 (25.6%) patients fulfilled the definition of stable chronic heart failure, and 1878 (48.4%) had suffered previous episodes of congestive heart failure (> 30 days before procedure). Among patients with chronic stable heart failure, the mean (SD) age was 73 (8) y, and 1397 (36%) were female. Table 1 reports the baseline characteristics of the whole cohort and of patients with and without heart failure. Country‐specific demographics can be found in online Supporting Information Table S2.

**Table 1:** Baseline characteristics of the entire cohort and stratified by the presence of heart failure diagnosis. Values are number (proportion) or median (IQR [range]).

	Total cohort	No heart failure	Heart failure
	n = 15,158	n = 11,278	n = 3880
Sex; female	5944 (39.2%)	4547 (40.3%)	1397 (36.0%)
Age; y			
40–59	681 (4.5%)	500 (4.4%)	181 (4.7%)
60–74	8252 (54.4%)	6556 (58.2%)	1696 (43.7%)
≥ 75	6224 (41.1%)	4221 (37.4%)	2003 (51.6%)
ASA physical status ≥ 3	8729 (57.6%)	5715 (50.7%)	3014 (77.7%)
eGFR; ml.min^‐1^.1.73 m^‐2^			
< 30 or dialysis	660 (4.4%)	347 (3.1%)	313 (8.1%)
30–60	3093 (20.4%)	1990 (17.6%)	1103 (28.4%)
≥ 60	11,086 (73.1%)	8708 (77.2%)	2378 (61.3%)
Diabetes mellitus			
Oral hypoglycaemic drugs	2326 (15.3%)	1678 (14.9%)	648 (16.7%)
Insulin	1315 (8.7%)	786 (7.0%)	529 (13.6%)
Ischaemic heart disease	3635 (24.0%)	1961 (17.4%)	1674 (43.1%)
Peripheral vascular disease	2984 (19.7%)	1885 (16.7%)	1099 (28.3%)
Stroke or TIA	1768 (11.7%)	1139 (10.1%)	629 (16.2%)
Active cancer	6998 (46.2%)	5399 (47.9%)	1599 (41.2%)
COPD	2091 (13.8%)	1413 (12.5%)	678 (17.5%)
Hypertension	11,077 (73.1)	7814 (69.3)	3263 (84.1%)
Type of surgery			
Low‐risk	1067 (7.0%)	650 (5.8%)	417 (10.7%)
Moderate‐risk	9977 (65.8%)	7615 (67.5%)	2362 (60.9%)
High‐risk	4113 (27.1%)	3012 (26.7%)	1101 (28.4%)
Functional status			
Independent	12,238 (80.7%)	9452 (83.8%)	2786 (71.8%)
Partially independent	2617 (17.3%)	1637 (14.5%)	980 (25.3%)
Fully dependent	302 (2.0%)	188 (1.7%)	114 (2.9%)
Pre‐operative NT‐proBNP, pg.ml^‐1^	171 (79–461 [0–92,418])	64 (39–95 [0–124])	337 (187–808 [0–92,418])
Revised cardiac risk index			
Low, ≤ 1 point	12,743 (84.1%)	10,482 (93.7%)	2261 (58.4%)
Moderate, 2 points	1697 (11.2%)	613 (5.5%)	1084 (28.0%)
High, ≥ 3 points	618 (4.1%)	90 (0.8%)	528 (13.6%)

eGFR, estimated glomerular filtration rate; TIA, transient ischaemic attack; COPD, chronic obstructive pulmonary disease; NT‐proBNP, N‐type pro brain natriuretic peptide.

Among patients with stable chronic heart failure, TTE had been requested by the attending physicians in 476/1101 patients (43.2%) within the 6 months before high‐risk surgery; 756/2362 (32.0%) before moderate‐risk surgery; and 148/417 (35.5%) before low‐risk surgery (Table 2). Patients who had pre‐operative TTE were younger, had more comorbidities (ASA physical status ≥ 3: ischaemic heart disease; history of stroke or transient ischaemic attack; COPD and hypertension) and more cardiovascular risk factors (higher RCRI score) than patients who did not have pre‐operative TTE. Furthermore, NT‐proBNP values were significantly higher in patients who had pre‐operative TTE than in patients without it (Table 2).

**Table 2:** Baseline characteristics of heart failure patients stratified by conduction of pre‐operative transthoracic echocardiography (TTE) 6 months before surgery. Values are number (proportion) or median (IQR [range]).

	No pre‐operative TTE	Pre‐operative TTE	p value
	n = 2500	n = 1380	
Sex; female	961 (38.4%)	436 (31.6%)	< 0.001
Age; y			
40–59	115 (4.6%)	104 (7.5%)	< 0.001
60–74	1174 (47.0%)	608 (44.1%)	< 0.044
≥ 75	1211 (48.4%)	668 (48.4%)	0.505
ASA physical status ≥ 3	1799 (72.0%)	1215 (88.0%)	< 0.001
eGFR; ml.min^‐1^.1.73 m^‐2^			0.889*
< 30 or dialysis	199 (8.0%)	114 (8.5%)	
30–60	715 (28.6%)	388 (29.1%)	
≥ 60	1545 (61.8%)	833 (62.4%)	
Diabetes mellitus			0.410*
Oral hypoglycaemic drugs	403 (16.1%)	245 (17.8%)	
Insulin	346 (13.8%)	183 (13.3%)	
Ischaemic heart disease	869 (34.8%)	805 (58.3%)	< 0.001
Peripheral vascular disease	687 (27.5%)	412 (29.9%)	0.118
Stroke or TIA	379 (15.2%)	250 (18.1%)	0.018
Active cancer	1023 (41.0%)	576 (41.7%)	0.633
COPD	369 (14.8%)	309 (22.4%)	< 0.001
Hypertension	2008 (80.3%)	1255 (90.9%)	< 0.001
Type of surgery			< 0.001*
Low‐risk	269 (10.8%)	148 (10.7%)	0.510
Moderate‐risk	1606 (64.2%)	756 (54.8%)	< 0.001
High‐risk	625 (25.0%)	476 (34.5%)	< 0.001
Pre‐operative NT‐proBNP; pg.ml^‐1^	317 (184–736 [0–73,046])	402 (204–1016 [10–92,418])	0.003
Revised cardiac risk index			< 0.001*
Low, ≤ 1 point	1610 (63.3%)	651 (47.3%)	< 0.001
Moderate, 2 points	601 (24.0%)	483 (35.1%)	< 0.001
High, ≥ 3 points	285 (11.4%)	243 (17.6%)	< 0.001

eGFR, estimated glomerular filtration rate; TIA, transient ischaemic attack; COPD, chronic obstructive pulmonary disease; NT‐proBNP, N‐type pro brain natriuretic peptide.

* p value for interaction.

Major adverse cardiovascular events at 30 days postoperatively occurred in 152/3880 (3.9%) and 167/11,278 (1.5%) patients with and without heart failure, respectively. Mortality at 30 days occurred in 135/3880 (3.5%) and 200/11,278 (1.8%) patients with and without heart failure, respectively. Severe in‐hospital complications with a Clavien‐Dindo classification ≥ 3 were observed in 546/3880 (14.1%) and 1036/11,278 (9.2%) patients with and without heart failure, respectively. Online Supporting Information Table S3 reports outcomes stratified by surgical risk.

After multivariable adjustment, stable chronic heart failure remained independently associated with 30‐day MACE (OR 2.04, 95%CI 1.59–2.60, p < 0.001); 30‐day mortality (OR 1.50, 95%CI 1.17–1.92, p = 0.001); and severe postoperative complications (Clavien‐Dindo classification ≥ 3, OR 1.47, 95%CI 1.30–1.66, p < 0.001) (Table 3). Online Supporting Information Table S4 reports the detailed results of the multivariable analyses.

**Table 3:** Associations between chronic stable heart failure and postoperative outcomes stratified by surgery risk. Values are adjusted OR (95%CI).

Type of surgery	Mortality	MACE	Severe complications
Low‐risk	4.05 (0.38–42.87)	5.76 (1.10–30.08)	1.77 (0.89–3.56)
Moderate‐risk	1.86 (1.29–2.66)	1.97 (1.40–2.76)	1.44 (1.21–1.71)
High‐risk	1.20 (0.85–1.69)	1.95 (1.33–2.84)	1.49 (1.23–1.78)

MACE, major adverse cardiovascular event.

In the 6 months before surgery, TTE had been requested in 1380 (35.6%) patients with stable chronic heart failure, with preserved left ventricular ejection fraction present in 760 (59.6%). Mildly reduced and reduced left ventricular ejection fraction were documented in 366 (28.7%) and 149 (11.7%) patients, respectively (Table 2).

Compared with stable chronic HFpEF, after adjustment for age and RCRI, stable chronic HFrEF was associated with MACE (adjusted OR 2.00, 95%CI 1.01–3.81, p = 0.045) but not with 30‐day mortality (OR 1.04, 95%CI 0.46–2.34, p = 0.925) or postoperative complications (OR 1.31, 95%CI 0.82–2.08, p = 0.257). No differences in MACE, 30‐day mortality and postoperative complications were observed in patients with HFmrEF compared with those with HFpEF.

## Discussion

The main findings from this large European sample are that one in four patients undergoing non‐cardiac surgery aged ≥ 65 y, or > 45 y with cardiovascular risk factors, fulfil the current definition for heart failure. Only one in three patients with stable chronic heart failure received pre‐operative TTE within 6 months of major non‐cardiac surgery as recommended by the European Society of Cardiology guidelines [10]. In addition, stable chronic heart failure was independently associated with MACE, mortality and severe postoperative complications after non‐cardiac surgery.

The subgroup analysis stratified by ejection fraction suggested that patients with HFrEF suffered from significantly more MACE than patients with HFmrEF or HFpEF.

We found that one in four patients in our study cohort fulfilled the current European Society of Cardiology definition of heart failure. Previous administrative data using ICD‐9 codes to define heart failure reported a prevalence in the order of 5–8% [9, 22]. While direct comparison with these figures is not possible due to the use of different definitions and different populations, the evidence suggests that a meaningful proportion of patients presenting for non‐cardiac surgery have heart failure. Given the high volume of non‐cardiac surgical procedures, heart failure in the peri‐operative setting represents a major health problem at a population level across Europe (online Supporting Information Table S2).

Even though the current European Society of Cardiology guidelines recommend performing TTE in patients with known heart failure in the 6 months before elective, high‐risk non‐cardiac surgery (class 1 recommendation) [10], only 43.2% of patients with heart failure undergoing high‐risk procedures received this investigation. It was performed even less frequently in patients with heart failure undergoing moderate‐risk procedures (32.5%), for whom a class 2 recommendation for pre‐operative TTE was issued [10]. In contrast, in a Veterans Affairs database in the USA, 97% of patients with heart failure had received left ventricular ejection fraction quantification within the requisite timeframe [9]. We cannot comment on the outcome impact of performing or not performing TTE in patients with heart failure before non‐cardiac surgery due to limited data. In summary, the limited use of pre‐operative TTE suggests a lack of awareness of the prognostic implications of stable chronic heart failure among peri‐operative physicians in Europe.

Using a large, American administrative dataset, Turrentine et al. identified newly diagnosed or worsening congestive heart failure as being independently associated with adverse postoperative events, especially cardiac arrest (3.5% in patients with heart failure vs. 0.3% in patients without) and 30‐day mortality (12.4% in patients with heart failure vs. 1% in patients without) [23]. Lerman et al. referred to two national Veterans Affairs databases and classified patients as having heart failure if they had at least one inpatient admission or at least two outpatient clinic visits with a diagnosis of heart failure as defined by ICD codes within 3 years of non‐cardiac surgery [9]. They also observed an association between heart failure and increased 90‐day mortality (5.5% in patients with heart failure vs. 1.2% in patients without heart failure; adjusted absolute risk difference 1.03%, 95%CI 0.91–1.15%; adjusted OR 1.67, 95%CI 1.57–1.76). Patients with symptomatic heart failure had a high mortality risk compared to patients without heart failure (adjusted OR 2.37, 95%CI 2.14–2.63). Our results confirm the prognostic association between heart failure and adverse outcomes (30‐day death, 30‐day MACE and in‐hospital complications) after non‐cardiac surgery.

A retrospective cohort study of 174 patients with heart failure undergoing moderate‐ or high‐risk non‐cardiac surgery detected a significant association between left ventricular ejection fraction < 30% and an increased incidence of postoperative cardiovascular complications [24]. The authors concluded that a reduced left ventricular ejection fraction was an independent risk factor for adverse postoperative outcomes after non‐cardiac surgery, specifically all‐cause mortality, myocardial infarction and decompensation of heart failure. Subsequently, Lerman et al. also reported that decreased left ventricular ejection fraction was a risk factor for increased postoperative mortality (adjusted OR 1.67, 95%CI 1.57–1.76) [9]. Our study used current definitions of reduced left ventricular ejection fraction (< 40%) and observed an increased incidence of MACE at 30 days after non‐cardiac surgery, but not an increase in postoperative mortality. The partially divergent findings may arise from different definitions of reduced left ventricular ejection fraction than that used in previous studies; different populations studied; and the limited number of deaths in our sample.

The strengths of our analysis include a large, pan‐European sample derived from a prospective international cohort, high follow‐up completeness (97.7%) and minimal missing data for model covariables (3.3%). We acknowledge some limitations. First, we opted to use the MACE definition of the parent study [11]. The Standardised Endpoints in Perioperative Medicine initiative recommended a MACE definition consisting of all‐cause death and myocardial infarction [25]. Since the relevant recommendations were published after completion of the MET‐REPAIR study, the MACE definition used here is not consistent with that advocated by the Standardised Endpoints in Perioperative Medicine initiative. Second, TTE was requested before surgery in a minority of patients with heart failure. While an interesting finding, the analysis of the outcome impact of the heart failure subtypes stratified by left ventricular ejection fraction may be underpowered. Further, half of the patients with heart failure who underwent high‐risk procedures did not receive pre‐operative TTE. While further evaluation of the consequences of this finding is warranted, it does suggest that the pre‐operative assessment of patients with heart failure undergoing non‐cardiac surgery is inconsistent with current best practice recommendations.

In conclusion, stable chronic heart failure affects a meaningful proportion of patients undergoing non‐cardiac surgery. Despite our identifying an association with adverse outcomes, only a minority of patients with heart failure planned for major surgical procedures were investigated using TTE. Better co‐ordination of care is needed before planned surgery in patients with heart failure to improve peri‐operative outcomes.

## Supporting information


**Appendix S1.** MET‐REPAIR Investigators.

**Table S1.** Selection of covariables for multivariable logistic regression and rationale for categorisation.
**Table S2.** Prevalence of heart failure in different countries.
**Table S3.** Incidence of outcomes in patients with heart failure stratified by surgical risk.
**Table S4.** Independent association of heart failure with outcomes.
