# Supplementary material for: Outcomes in patients with chronic heart failure undergoing non‐cardiac surgery: a secondary analysis of the METREPAIR international cohort study*
Source: Anaesthesia. 2025 Apr 15;80(8):927–34. doi: 10.1111/anae.16607 (PMC12256159; doi:10.1111/anae.16607)
Supplement: Supplementary file 1 — Appendix S1. MET‐REPAIR Investigators. [file ANAE-80-927-s001.docx]

**Appendix S1. Full list of the MET-REPAIR Investigators**

1. **List of National Coordinators**

**Germany** - Giovanna **Lurati Buse**

**Italy**- Savino **Spadaro**

**Spain**- Purificación **Matute**

**Romania**- Daniela **Ionescu**

**Switzerland**- Daniel **Bolliger**

**Poland**- Wojciech **Szczeklik**

**Turkey**- Sanem Cakar **Turhan**

**Netherlands**- Judith **van** **Waes**

**Portugal**- Filipa **Lagarto**

**Greece**- Kassiani **Theodoraki**

**United** **Kingdom**- Simon J **Howell**

**Sweden**- Anil **Gupta**

**Belgium**- Stefan **De Hert**

**Russia**- Alexey **Ovezov**

**Republic of North Macedonia**- Biljana **Kuzmanovska**

**Croatia**- Stjepan **Barisin**

**Slovenia**- Peter **Poredos**

**Bulgaria**- Daniela **Arabadzhieva**

**Serbia**- Dragana **Unic**-**Stojanovic**

**Austria**- Edith **Fleischmann**

**France**- Claude **Meistelman**

**Ireland**- Donal J **Buggy**

**Malta**- Paul **Calleja**

**Kosovo**- Antigona **Hasani**

1. **Collaborators List: MET-REPAIR by country**

Germany

- Universitätsklinikum Düsseldorf: Sebastian **Roth**, René **M’Pembele**, Johannes **Nienhaus**, Alexandra **Stroda**, Theresa **Tenge**, Anna **Kirkopoulos**, Ragnar **Huhn,** Detlef **Kindgen-Milles,** Giovanna **Lurati Buse**
- Hannover Medical School: Cornelia **Schultze**, Nele **Verbarg**, Christian **Gehrke**, Anna Katharina **Klemann**, Friederike **Hagebölling,** Hans-Jörg **Gillmann**
- University Hospital Marburg: Svenja **Albrecht**, Jakob **Stroeder**, Ann-Kristin **Schubert,** Hinnerk **Wulf**
- Heidelberg University Hospital: Florian **Espeter**, Benedikt **Russe**, Jan **Larmann**, Markus A. **Weigand**
- University Hospital Knappschaftskrankenhaus Bochum: Lars **Bergmann**, Matthias **Unterberg**
- Marien Hospital Herne, Ruhr University Bochum: Petra **Bischoff**
- Universitätsklinikum Augsburg: Raphael **Pirzer**, Patric Rene **Rach**, Klaus **Ott**
- University Hospital Muenster: Alexander **Zarbock**
- University Hospital RWTH Aachen: Ana **Kowark**
- University Hospital Bonn: Claudia **Neumann**, Bahareh **Marchand**
- Jena University Hospital: Christoph **Sponholz**
- Helios Klinik Schkeuditz: Henrik **Rueffert**
- University Witten / Herdecke - Hospital Center Merheim Cologne: Mira **Kramer**
- University Hospital Frankfurt: Simone **Lindau**, Patrick **Meybohm,** Kai **Zacharowski**
- Universitätsklinikum Gießen, Justus-Liebig-Universität Gießen: Götz **Schmidt**, Christian **Koch**

Italy

- ASST Settelaghi – Ospedale di Circolo e Fondazione Macchi, Varese: Melissa **Carollo**, Cecilia **Novazzi**, Fiorenza **Toso**, Alessandro **Bacuzzi**
- University of Ferrara: Savino **Spadaro**
- University Hospital Varese: Luca Guzzetti
- Arcispedale Sant'Anna Ferrara: Riccardo **Ragazzi**, Carlo Alberto **Volta**, Francesco **De Giorgi**
- Azienda USL Di Bologna: Barbara **Bacer**, Antonio **Federico**
- ASST Santi Paolo e Carlo: Davide **Chiumello**
- Asuiud: Luigi **Vetrugno**
- Ospedale San Raffaele IRCCS: Alberto **Castella**
- University of Perugia Hospital Santa Maria della Misericordia: Simonetta **Tesoro**
- Hospital of Foggia: Antonella **Cotoia**
- Azienda Ospedaliero universitaria di Parma: Elena **Bignami**
- Arnas Garibaldi: Agrippino **Bellissima**
- University Hospital Policlinico P. Giaccone: Andrea **Cortegiani**
- Azienda Sanitaria Universitaria Integrata di Trieste: Marco **Crisman**
- ASST Spedali Civili Brescia: Arturo **Toninelli**
- University of Salerno-AOU San Giovanni di Dio e Ruggi D'Aragona: Ornella **Piazza**
- University of Foggia: Lucia **Mirabella**
- ASL CN1: Matteo **Bossolasco**
- FPO IRCCS CANDIOLO: Francesco **Bona**
- Fondazione Istituto “G.Giglio” of Cefalù
- Fondazione poliambulanza

Spain

- Hospital Clínic. UB. Barcelona: Juan Manuel **Perdomo**, Miquel **Coca-Martinez**, Albert

**Carramiñana,** Purificación **Matute**

- Hospital de la Santa Creu i Sant Pau: Marta **Giné Servén**, Astrid **Batalla** **González**, José Maria

**Gil Sánchez**

- Hospital Universitario de Gran Canaria Doctor Negrin: Ángel **Becerra**-**Bolaños**, Aurelio

**Rodríguez-Pérez**

- Corporacio sanitaria Parc Tauli: Anna **Artigas Soler**, Morena **Basso**, Anna **Peig Font**
- Fundacion Puigvert: Diana **Vernetta**
- Hospital Universitario La Princesa: Julia **Hernando Santos**, Enrique **Alday Muñoz**
- University Hospital of Guadalajara: Mercedes **Cabellos Olivares**
- Hospital Santa Maria: Gregorio **Marco**
- Hospital Universitario Lucus Augusti: Maria **Bermudez Lopez**
- Hospital Universitari Doctor Peset: Javier **Barrio**
- Hospital Arnau De Vilanova–Lliria: María Isabel **Forés**, Estefanía **Boix**
- Hospital Universitario Príncipe de Asturias: Mercedes **Ayuso**
- Ramon y Cajal Hospital
- Complejo Hospitalario de Mérida

Romania

- Central Military University Emergency Hospital From Romania: Bogdan Sorel **Petre**, Ioana

Sorina **Oprea**, Mihai Dan **Latiș**

- Iuliu Hatieganu University of Medicine and Pharmacy,Cluj-Napoca: Daniela **Ionescu**
- University Hospital Bucharest: Dan **Corneci**
- Regional Institute of Gastroenterology and Hepatology "Prof Dr Octavian Fodor” Cluj

Napoca: Simona **Margarit**, Horatiu **Vasian**

- Fundeni Clinical Institute: Dana **Tomescu**
- Constanta County Emergency Clinical Hospital: Iulia **Cîndea**
- Emergency county hospital cluj: Dan Sebastian **Dirzu**
- Spital Clinic Judetean de Urgenta Targu Mures: Sanda-Maria **Copotoiu**
- Prof Dr Gerota Hospital: Alida **Moise**
- Emergency Institute for CardioVascular Diseases Prof. Dr. C.C.Iliescu: Serban **Bubenek-Turconi**, Liana **Valeanu**
- Elias University Emergency Hospital

Switzerland

- Kantonsspital St. Gallen: Patrick Mark **Wanner**, Mirjana **Djurdjevic**, Sandra **Nuth,** Miodrag **Filipovic**
- Basel University Hospital: Esther **Seeberger**, Nicolai **Goettel**, Firmin **Kamber,** Eckhard **Mauermann,** Daniel **Bolliger,** Frederique **Chammartin**
- Kantonsspital Winterthur: Michael Thomas **Ganter**, Thomas Jan **Gerber**, Daniela **Schneebeli**
- University Hospital Zurich: Andreas **Pregernig,** Beatrice **Beck-Schimmer**
- Hôpital du Valais: Sina **Grape**
- Geneva University Hospitals: Simon **Tomala**, Bernardo **Bollen Pinto**

Poland

- Pomeranian Medical University: Maciej **Żukowski**, Małgorzata **Zegan-Barańska**, Igor **Karolak,** Katarzyna **Kotfis**
- Jagiellonian University Medical College: Wojciech **Szczeklik**
- Medical University of Silesia: Lukasz **Krzych**, Szymon **Czajka**
- St John Grande Hospital: Dorota **Studzińska**
- Heliodor Swiecicki Clinical Hospital at Poznan University of Medical Sciences: Anna **Kluzik**,

Tomasz **Koszel**

- The University Hospital in Krakow: Izabela **Pabjańczyk**
- St. Raphael Hospital: Anna **Gajdosz**
- Medical University of Białystok

Turkey

- Ankara University Medical School: Suheyla **Karadag Erkoc**, Basak Ceyda **Meco,** Sanem **Cakar Turhan**
- Istanbul University Istanbul Faculty of Medicine: Ahmet Kemalettin **Koltka**, Muserref Beril **Dincer**
- Ufuk University Faculty of Medicine: Perihan **Ekmekçi**
- Kocaeli Derince Training and Research Hospital: Kemal Tolga **Saracoglu**
- Suleyman Demirel University School of Medicine: Filiz **Alkaya** **Solmaz**
- Ankara University School of Medicine Cebeci Hospital: Menekse **Ozcelik**
- Selcuk University Faculty of Medicine: Oguzhan **Arun**
- Istanbul University- Cerrahpasa, Cerrahpasa Faculty of Medicine: Ozlem **Korkmaz Dilmen**
- Hacettepe University Hospital

Netherlands

- Amsterdam UMC, location AMC, Amsterdam: Benedikt **Preckel**, Markus W. **Hollmann**
- University Medical Center Utrecht: Yannick **Hazen,** Judith **van Waes**
- Martini General Hospital Groningen: Hans Donald **de Boer**
- University Medical Center Groningen (UMCG): Anne **Epema**
- Maasstad Hospital: Seppe **Koopman**
- Erasmus MC: Felix **Van Lier**

Portugal

- Hospital Beatriz Ângelo: Rita **Pinto**, André **Carrão**, Daniel **Ribeiro,** Filipa **Lagarto**
- Centro Hospitalar São João: Joana **Mourão**
- Centro Hospitalar do Baixo Vouga: Miguel **Coelho**
- Centro Hospitalar de Vila Nova de Gaia / Espinho; Nuno **Losa**
- Hospital de Santo Espírito da Ilha Terceira, E.P.E.R.: Nuno **Santos**, Luis **Cabral**
- Centro Hospitalar Entre Douro e Vouga, EPE: Diana **Afonso**
- Hospital Central do Funchal: Sérgio **Zenha**
- Hospital de Sta. Marta, CHLC: Cristina **Ramos**
- Hospital de Braga: Carla **Hipólito**
- Hospital Cuf Santarém

Greece

- General Hospital of Athens "G. Gennimatas": Maria **Vasilaki**, Antonia **Andreeva**
- General Hospital of Thessaloniki "Ippokratio": Donika **Zaimi**
- Tzaneio General Hospital of Piraeus: Athanasios **Chalkias**
- University Hospital of Patras: Maria **Spyraki**
- Aretaieion University Hospital: Martina **Rekatsina,** Kassiani **Theodoraki**
- Ahepa University Hospital: Georgia **Tsaousi**
- General Hospital of Athens "Alexandra"
- General Hospital of Chalkidiki

United Kingdom

- Wrightington, Wigan and Leigh NHS Foundation Trust: Anthony **Short**
- Guys and St Thomas NHS Foundation Trust: Sonja **Meier**
- Royal Surrey NHS Foundation Trust: Thumuluru Kavitha **Madhuri**
- Royal Bolton Hospital: Scott **Latham**
- York Teaching Hospital: James **Knock**
- The Royal Oldham Hospital - The Pennine Acute Hospitals NHS Trust: Andrew **Drummond**
- Queen Victoria Hospital: Fiona **Ramsden**
- Yeovil Hospital: Agnieszka **Kubisz-Pudelko**
- Royal Bournemouth Hospital: James **Walker**
- Nottingham University Hospital: Queen’s Medical Centre: Iain **Moppett**
- St James' University Hospital: Louise **White**
- University of Leeds: Simon J. **Howell**
- Stockport NHS Foundation Trust: Matthew **Jackson**
- Poole Hospital NHS Foundation Trust: Henrik **Reschreiter**
- Musgrove Park Hospital: Richard **Innes**
- NHS Ayrshire & Arran University Hospital Cross house
- King's College Hospital NHS Foundation Trust
- Salisbury NHS Foundation Trust
- Cwm Taf Morgannwg University Health Board

Sweden

- Linköping University Hospital: Michelle **Chew**
- Karolinska University Hospital and Karolinska Institutet: Sigridur **Kalman,** Anil **Gupta**
- Sundsvall Hospital: Jakob **Wallden**
- Karolinska Institute Physiology and Pharmacology: Anna **Schening**
- Uppsala University Hospital: Lina **Jonikaite**
- Uppsala University: Anna **Enlund**

Belgium

- Ghent University Hospital: Luc **De Baerdemaeker,** Stefan **de Hert**
- Antwerp University Hospital: Stuart **Morrison**
- University Hospitals Leuven: Steffen **Rex**
- CHU Saint-Pierre: Alexandros **Alexis**

Russia

- P.A. Herzen Moscow Cancer Research Institute: Viktoria E. **Khoronenko**
- Moscow Regional Research Clinical Institute: Alexey **Ovezov**
- Privolzhskiy District Medical Center: Vladislav **Belskii**, Kseniya **Kaznacheeva**
- Krasnoyrask Regional Clinical Hospital; Professor V.F. Voino-Yasenetsky Krasnoyarsk State Medical University: Alexey **Gritsan**

Republic of North Macedonia

- Clinic for Anesthesiology, Reanimation and Intensive Care medicine: Biljana **Kuzmanovska**
- GOB 8-mi Septemvri, Skopje: Liljana **Malinovska-Nikolovska**

Croatia

- UK Dubrava, J.J. Strossmayer University of Osijek, Faculty of Medicine: Stjepan **Barisin**

Slovenia

- University Medical Centre Ljubljana: Peter **Poredos**

Bulgaria

- University Hospital Kaneff Ruse: Daniela **Arabadzhieva**

Serbia

- Cardiovascular Institute Dedinje: Dragana **Unic-Stojanovic**
- Clinical Center Nis, Serbia: Mladjan **Golubović**

Austria

- Medical University of Vienna: Edith **Fleischmann**
- Klinikum Wels Grieskirchen: Oskar **Kotzinger**

France

- Military Teaching Hospital Clermont Tonnerre : Marc **Danguy Des Deserts**
- Clinique du Millénaire : Nicolas **Ducrocq**

Ireland

- Mater University Hospital, University College Dublin: Donal J **Buggy**, Jean François **Bonnet**, Barbara **Cusack**

Malta

- Mater Dei Hospital: Paul **Calleja**

Kosovo

- University of Prishtina, Faculty of Medicine: Antigona **Hasani**, Rajmonda **Nallbani**

**Management Team**

European Society of Anaesthesiology and Intensive Care, Brussels:

Sylvia Daamen, Benoit Plichon, Pierre Harlet, Slama Farsi, Saman Homayun Sepehr and David

Espinosa
