# Supplementary material for: Outcomes in patients with chronic heart failure undergoing non‐cardiac surgery: a secondary analysis of the METREPAIR international cohort study*
Source: Anaesthesia. 2025 Apr 15;80(8):927–34. doi: 10.1111/anae.16607 (PMC12256159; doi:10.1111/anae.16607)
Supplement: Supplementary file 2 — Table S1. Selection of covariables for multivariable logistic regression and rationale for categorisation. Table S2. Prevalence of heart failure in different countries. Table S3. Incidence of outcomes in patients with heart failure stratified by surgical risk. Table S4. Independent association of heart failure with outcomes. [file ANAE-80-927-s002.docx]

**Table S1. Selection of covariables for multivariable logistic regression and rationale for categorisation**

| **Confounder** |  | **Rationale for categorisation** |
| --- | --- | --- |
| Age | Years, continuous | na |
| Sex | Male vs female | na |
| ASA | ≥III vs I-II | Chieh Yang Koo et al. A Meta-analysis of the Predictive Accuracy of Postoperative Mortality Using the American Society of Anesthesiologists’ Physical Status Classification System. *World J Surg* 2015; **39**:88–103. |
| Surgery risk class | Low vs intermediate vs high | Kristensen SD et al. 2014 ESC/ESA Guidelines on non-cardiac surgery: cardiovascular assessment and management: The Joint Task Force on non-cardiac surgery: cardiovascular assessment and management of the European Society of Cardiology (ESC) and the European Society of Anaesthesiology (ESA). *Eur J Anaesthesiol* 2014; **31**: 517-73. |
| Surgical urgency | Elective vs semi-elective (2-7d) vs urgent (<=1d) | Adapted from: The Vascular Events in Noncardiac Surgery Patients Cohort Evaluation (VISION) Study Investigators Association Between Postoperative Troponin Levels and 30-Day Mortality Among Patients Undergoing Noncardiac Surgery, *JAMA* 2012; **307**(21):2295-2304. |
| Hx of heart failure | LVEF <40%, 40-50%, >50%, not quantified | Ponikowski P, et al, 2016 ESC Guidelines for the diagnosis and treatment of acute and chronic heart failure: The Task Force for the diagnosis and treatment of acute and chronic heart failure of the European Society of Cardiology (ESC) Developed with the special contribution of the Heart Failure Association (HFA) of the ESC. *Eur Heart J* 2016; **37**(27):2129-2200. |
| Hx of PAVD | Yes/no | na |
| Hx of stroke/TIA | Yes/no | na |
| Hx of diabetes | No vs noninsulin-dependent vs insulin-dependent | Adapted from Lee TH, et al. Derivation and prospective validation of a simple index for prediction of cardiac risk of major noncardiac surgery. *Circulation* 1999; **100** (10): 1043-1049. |
| Renal failure | I-II vs ≥III vs  dialysis | Kidney Disease: Improving Global Outcomes (KDIGO) CKD Work Group. KDIGO 2012 Clinical Practice Guideline for the Evaluation and Management of Chronic Kidney Disease. *Kidney inter* 2013; **3**: 1–150. |
| COPD | Yes/no | na |
| Cancer surgery | Yes/no | na |
| Hx of CAD | Yes/no | na |

ASA, American Society of Anesthesiologists; d, days; Hx, history; LVEF, left ventricular ejection fraction; PAVD, peripheral arterial disease; TIA, transitory ischemic attack; COPD, chronic pulmonary obstructive disease; CAD, coronary artery disease.

**Table S2.** Prevalence of heart failure in different countries. Values are number (proportion).

|  | **Total cohort**  **n = 15,157** | **No HF**  **n = 11,277** | **HF (all)**  **n = 3,880** | **HFrEF**  **(n, %)** | **HFmrEF**  **(n, %)** | **HFpEF**  **(n, %)** |
| --- | --- | --- | --- | --- | --- | --- |
| USA | 192 | 188 (97.9%) | 4  (2.1%) | 1  (25.0%) | 1  (25.0%) | 0  (0%) |
| Russia | 361 | 286 (79.2%) | 75 (20.8%) | 3  (4.0%) | 12  (16.0%) | 45  (60.0%) |
| Greece | 580 | 521 (89.8%) | 59 (10.2%) | 4  (6.8%) | 31  (52.5%) | 10  (16.9%) |
| Netherlands | 625 | 561 (89.8%) | 64 (10.2%) | 9  (14.1%) | 9  (14.1%) | 4  (6.3%) |
| Belgium | 410 | 366 (89.3%) | 44 (10.7%) | 4  (9.1%) | 10  (22.7%) | 15  (34.1%) |
| France | 99 | 96 (97.0%) | 3  (3.0%) | 0  (0%) | 0  (0%) | 2  (66.7%) |
| Spain | 1691 | 1314 (77.7%) | 377 (22.3%) | 11  (2.9%) | 10  (2.7%) | 32  (8.5%) |
| Italy | 2104 | 1656 (78.7%) | 448 (21.3%) | 26  (5.8%) | 70  (15.6%) | 115 (25.7%) |
| Romania | 1172 | 815 (69.5%) | 357 (30.5%) | 13  (3.6%) | 89  (24.9%) | 164 (45.9%) |
| Switzerland | 940 | 395 (42.0%) | 545 (58.0%) | 11  (2.0%) | 19  (3.5%) | 128 (23.5%) |
| Austria | 101 | 92 (91.1%) | 9  (8.9%) | 1  (1.0%) | 1  (1.0%) | 0  (0%) |
| United Kingdom | 464 | 430 (92.7%) | 34  (7.3%) | 2  (5.9%) | 2  (5.9%) | 12  (35.3%) |
| Sweden | 444 | 413 (93.0%) | 31  (7.0%) | 4  (12.9%) | 3  (9.7%) | 4  (12.9%) |
| Poland | 799 | 528 (66.1%) | 271 (33.9%) | 6  (2.2%) | 11  (4.1%) | 16  (5.9%) |
| Germany | 2,872 | 1,735 (60.4%) | 1,137 (39.6%) | 28  (2.5%) | 36  (3.2%) | 144 (12.7%) |
| Turkey | 921 | 803 (87.2%) | 118 (12.8%) | 17  (14.4%) | 40  (33.9%) | 34  (28.8%) |
| Portugal | 607 | 458 (75.5%) | 149 (24.5%) | 3  (2.0%) | 3  (2.0%) | 10  (6.7%) |
| Ireland | 25 | 13 (52.0%) | 12 (48.0%) | 0  (0%) | 1  (8.3%) | 0  (0%) |
| Malta | 63 | 55 (87.3%) | 8  (12.7%) | 0  (0%) | 0  (0%) | 3  (37.5%) |
| Bulgaria | 107 | 49 (45.8%) | 58 (54.2%) | na | na | na |
| Serbia | 102 | 89 (87.3%) | 13 (12.7%) | 4  (30.8%) | 7  (53.8%) | 1  (7.7%) |
| Kosovo | 51 | 46 (90.2%) | 5  (9.8%) | 0  (0%) | 1  (20.0%) | 4  (80.0%) |
| Croatia | 153 | 140 (91.5%) | 13  (8.5%) | 0  (0%) | 3  (23.1%) | 4  (30.8%) |
| Slovenia | 112 | 88 (78.6%) | 24 (21.4%) | 1  (4.2%) | 3  (12.5%) | 11  (45.8%) |
| North Macedonia | 162 | 140 (86.4%) | 22 (13.6%) | 2  (9.1%) | 4  (18.2%) | 2  (9.1%) |

HF, heart failure; HFrEF, heart failure with reduced ejection fraction; HFmrEF, heart failure with mildly reduced ejection fraction; HFpEF, heart failure with preserved ejection fraction; na, not applicable. Data on ejection fraction are shown as provided by performed transthoracic echocardiography (TTE). TTE was not performed in all HF cases.

**Table S3. Incidence of outcomes in patients with heart failure stratified by surgical risk. Values are number (proportion)**

| **30-day mortality** | | | | | | |
| --- | --- | --- | --- | --- | --- | --- |
|  | **Total cohort**  **n = 15,155** | **No heart failure**  **n = 11,276** | **Heart failure (all)**  **n = 3,880** | **HFpEF**  **n = 759** | **HFmrEF**  **n = 366** | **HFrEF**  **n = 150** |
| Low-risk surgery | 6 (0.03%) | 2 (0.02%) | 4 (0.1%) | 1 (0.03%) | 1 (0.3%) | 0 (0%) |
| Intermediate-risk surgery | 148 (1.0%) | 82 (0.7%) | 66 (1.7%) | 14 (0.4%) | 4 (1.1%) | 3 (0.1%) |
| High-risk surgery | 181 (1.2%) | 116 (1.0%) | 65 (1.7%) | 18 (0.5%) | 8 (2.2%) | 5 (0.1%) |
| **30-day MACE** | | | | | | |
|  | **Total cohort**  **n = 15,155** | **No heart failure**  **n = 11,276** | **Heart failure (all)**  **n = 3,880** | **HFpEF**  **n = 759** | **HFmrEF**  **n = 366** | **HFrEF**  **n = 150** |
| Low-risk surgery | 13 (0.1%) | 3 (0.02%) | 10 (0.3%) | 3 (0.1%) | 1 (0.03%) | 1 (0.03%) |
| Intermediate-risk surgery | 167 (1.1%) | 90 (0.8%) | 77 (2.0%) | 14 (0.4%) | 4 (0.1%) | 5 (0.1%) |
| High-risk surgery | 139 (1.0%) | 74 (0.7%) | 65 (1.7%) | 18 (0.5%) | 8 (0.2%) | 7 (0.2%) |
| **Severe in-hospital complications (CDC ≥ 3)** | | | | | | |
|  | **Total cohort**  **n = 15,155** | **No heart failure**  **n = 11,276** | **Heart failure (all)**  **n = 3,880** | **HFpEF**  **n = 759** | **HFmrEF**  **n = 366** | **HFrEF**  **n = 150** |
| Low-risk surgery | 42 (0.3%) | 18 (0.2%) | 24 (0.6%) | 4 (0.1%) | 6 (0.2%) | 1 (0.03%) |
| Intermediate-risk surgery | 784 (5.2%) | 517 (4.6%) | 267 (6.9%) | 54 (1.4%) | 13 (0.3%) | 8 (0.2%) |
| High-risk surgery | 756 (5.0%) | 501 (4.4%) | 255 (6.6%) | 55 (1.4%) | 27 (0.7%) | 19 (0.5%) |

CDC, Clavien Dindo Classification; HF, heart failure; EF, ejection fraction; HFpEF, heart failure with preserved ejection fraction; HFmrEF, heart failure with mildly reduced ejection fraction; HFrEF, heart failure with reduced ejection fraction.

**Table S4**. **Independent association of heart failure with outcomes**

|  | **30-day mortality**  **Adjusted OR**  **(95% CI)** | **30-day MACE**  **Adjusted OR**  **(95% CI)** | **Severe complications**  **Adjusted OR**  **(95% CI)** |
| --- | --- | --- | --- |
|  |  |  |  |
| Age, y | 1.54 (1.28 – 1.86) | 1.40 (1.16 – 1.69) | 1.01 (0.92 – 1.10) |
| Sex, female | 0.74 (0.58 – 0.94) | 0.93 (0.73 – 1.19) | 0.83 (0.74 – 0.93) |
| ASA physical status ≥ 3 | 1.72 (1.28 – 2.30) | 1.17 (0.87 – 1.56) | 1.56 (1.37 – 1.77) |
| Preoperative functional status | 2.01 (1.65 – 2.45) | 1.27 (1.02– 1.59) | 1.27 (1.13 – 1.42) |
| Planned type of surgery (high-risk) | 2.80 (2.26 – 3.46) | 2.03 (1.65 – 2.61) | 2.22 (2.00 – 2.45) |
| eGFR, ml.min^-1^.1.73^-2^ | 0.69 (0.57 – 0.83) | 0.65 (0.55 – 0.78) | 0.83 (0.75 – 0.91) |
| Active cancer | 2.03 (1.57 – 2.63) | 1.17 (0.91 – 1.45) | 1.93 (1.71 – 2.18) |
| Hypertension | 0.76 (0.58 – 1.01) | 0.97 (0.71 – 1.31) | 0.94 (0.82 – 1.07) |
| Ischemic heart disease | 1.04 (0.79 – 1.35) | 1.40 (1.08 – 1.82) | 0.94 (0.82 – 1.08) |
| COPD | 1.13 (0.84 – 1.51) | 1.33 (0.99 – 1.77) | 1.24 (1.07 – 1.43) |
| Peripheral vascular disease | 0.99 (0.73 – 1.33) | 1.21 (0.91 – 1.60) | 1.07 (0.92 – 1.24) |
| Stroke or TIA | 0.99 (0.71 – 1.40) | 1.10 (0.79 – 1.52) | 1.13 (0.95 – 1.33) |
| Chronic heart failure | 1.50 (1.17 – 1.92) | 2.04 (1.59 – 2.60) | 1.47 (1.30 – 1.66) |

Y, years; ASA, American Society of Anesthesiology; eGFR, estimated glomerular filtration rate; COPD, chronic obstructive pulmonary disease; TIA, transitory ischemic attack; MACE, major adverse cardiovascular event; OR, odds ration; CI, 95% confidence interval.
